# Fast media optimization for mixotrophic cultivation of *Chlorella vulgaris*

**DOI:** 10.1038/s41598-019-55870-9

**Published:** 2019-12-17

**Authors:** Valerie C. A. Ward, Lars Rehmann

**Affiliations:** 1 Department of Chemical and Biochemical Engineering, University of Western Ontario, 1151 Richmond St., London, Ontario N6A 3K7 Canada; 2 Department of Chemical Engineering, University of Waterloo, 200 University Ave. W, Waterloo, Ontario N2L 3G1 Canada

**Keywords:** Chemical engineering, Applied microbiology

## Abstract

Microalgae can accumulate large proportions of their dry cell weight as storage lipids when grown under appropriate nutrient limiting conditions. While a high ratio of carbon to nitrogen is often cited as the primary mode of triggering lipid accumulation in microalgae, fast optimization strategies to increase lipid production for mixotrophic cultivation have been difficult to developed due to the low cell densities of algal cultures, and consequently the limited amount of biomass available for compositional analysis. Response surface methodologies provide a power tool for assessing complex relationships such as the interaction between the carbon source and nitrogen source. A 15 run Box-Behnken design performed in shaker flasks was effective in studying the effect of carbon, nitrogen, and magnesium on the growth rate, maximum cell density, lipid accumulation rate, and glucose consumption rate. Using end-point dry cell weight and total lipid content as assessed by direct transesterification to FAME, numerical optimization resulted in a significant increase in lipid content from 18.5 ± 0.76% to 37.6 ± 0.12% and a cell density of 5.3 ± 0.1 g/L to 6.1 ± 0.1 g/L between the centre point of the design and the optimized culture conditions. The presented optimization process required less than 2 weeks to complete, was simple, and resulted in an overall lipid productivity of 383 mg/L·d.

## Introduction

Lipid production in oleaginous microorganisms has been under investigation as a potential source of renewable lipids for biodiesel synthesis^1–5^. Currently, biodiesel is predominately synthesized from vegetables oils or waste fats which has created a global increase in the demand and ergo the price of these oils^6,7^. Accordingly, production of second-generation biodiesel from non-edible oils such as those derived from oleaginous microbes is of growing interest to the biodiesel industry^8–11^.

Almost all microbial species are capable of production of lipids, however, those capable of accumulating over 20% of their dry weight as lipids are designated oleaginous microorganisms^12^. Those species which accumulate such significant portions generally use these lipids as an alternative energy storage molecule in the form of triacylglycerides (TAGs)^7^. TAGs extracted from these organisms can be upgraded into fatty acid methyl esters (FAME); the most common form of biodiesel, using traditional transesterification processes. Microalgae have received a significant amount of attention as oleaginous microbes due to their ability to accumulate lipids from inorganic carbon (CO_2_) using photosynthesis^3^. However, low growth rates and lipid productivity has increased interest in the heterotrophic or mixotrophic (combined heterotrophic and phototrophic growth) cultivation^13–19^. Supplementation with organic carbon like glucose or acetate can greatly augment the growth rate and lipid productivity of these strains, particularly when grown under mixotrophic conditions^5,20^. Overall conversion of glucose to lipid has been relatively low for most single celled oils (SCOs) between 0.12–0.17 g/g compared to the theoretical maximum of 0.3 g/g for *Rhodosporidium toruloides*^21^. A high yield; 0.298 g/g glucose, has been obtained for the microalgae *Chlorella protothecoides* when using a mixed mode photosynthetic-heterotrophic cultivation process^22^. However, much like other microorganisms grown on organic carbon sources, use of carbon derived from waste sources like lignocellulosic biomass, waste water, pyrolytic sugars or other waste streams will ultimately be preferable^19,23^.

While reports of the effects of media composition on the heterotrophic cultivation of microalgae have been steadily increasing, many questions remain as to the complex relationship between carbon and nitrogen on the metabolism of these species. While it is known that lipid production increases with nitrogen or phosphorus limitation, excessive restriction of these substrates will also affect maximum cell density and consequently overall volumetric lipid productivity (g/L·d)^24^. While a high ratio of carbon to nitrogen (C/N) is often cited as the main method for triggering lipid production, limitation of nitrogen also limits the overall growth of the culture^8,24^.

In order to further elucidate the effects of nitrogen and carbon as independent factors and the effects of their interaction, response surface methodology was used. Three major nutrients, glucose, sodium nitrate, and magnesium sulfate was conducted using a Box-Behnken design. Multiple responses were analyzed including: glucose consumption, lipid accumulation, growth rate, end point dry cell weight and lipid content in mixotrophic shaker flask cultivations and the optimal conditions for high biomass and lipid productivity were predicted and confirmed.

## Materials and Methods

### Strain and culture conditions

*Chlorella vulgaris* strain UTEX 2714 was purchased from The Culture Collection of Algae at the University of Texas Austin. The culture was maintained as an actively growing culture in liquid media using aseptic technique in 150 mL Tris-acetate-phosphate (TAP) media (20 mM Tris base, 1.58 mM K_2_HPO_4_, 2.4 mM KH_2_PO_4_, 7.0 mM NH_4_Cl, 0.83 mM MgSO_4_, 0.34 mM CaCl_2_, 1 mL/L glacial acetic acid, and 1 mL/L of Hutner’s trace elements solution^25^) at pH 6.5 in 500 mL shaker flasks. All cultures were grown and maintained at 25 °C at 150 rpm under cyclic illumination consisting of 16 h on: 8 h off (100 μmol m^2^/s).

### Box-Behnken design

The effect of nitrate, glucose, and magnesium on various culture parameters were measured using a Box-Behnken design (BBD). This design was chosen due to poor predictability at the factor extremes when using a central composite design (data not shown) and in order to reduce the number of runs required. The factors and concentrations tested in this study are summarized in Table 1 and the design was replicated twice with 3 center point repeats.

**Table 1:** Coded and uncoded concentrations of media components used in the Box-Behnken design.

		Coded Levels and Concentration		
Component	Label	−1	0	1
Glucose (g/L)	*x*_1_	2	11	20
NaNO_3_ (g/L)	*x*_2_	0.225	1.375	2.55
MgSO_4_ · 7H_2_O (g/L)	*x*_3_	0.25	1.375	2.5

Coded and uncoded concentrations of media components used in the Box-Behnken design. Media components studied at different levels were added to a basal media according to the BBD and autoclaved prior to inoculation. The basal media contained 20 mM Tris base, 1.74 g/L KH_2_PO_4_, 0.04 g/L CaCl_2_ 2H_2_O, and 1 mL/L of Hutner’s Trace element solution and was adjusted to a pH of 6.8 using 5 M NaOH. A seed culture was prepared in TAP media 48 h prior to inoculation of the BBD media and cells were harvested aseptically by sterile centrifugation and resuspended in an equal volume of sterile water in order to minimize carryover of residual acetate and ammonium from the preculture. BBD cultures were prepared in a working volume of 75 mL in a 250 mL shaker flasks and inoculated with 1% v/v of the centrifuged seed culture.

### Analytical procedures

Samples of the culture were taken every 12 hours after 24 of incubation for up to 192 h. Samples were processed for several measurements according to the following general procedure: first optical density was measured followed by centrifugation and measurement of glucose concentration in the supernatant. The cell pellet was then bleached and used to measure lipid accumulation using the Nile Red assay. At the culture end point (glucose consumption ceased), the endpoint dry cell weight was measured by centrifugation and washing of the cell pellet followed by lyophilization. The freeze-dried cells were used for total lipid determination using a direct transesterification protocol developed by NREL. Specific procedures documented below.

### Optical density and growth rate

Cell suspensions were appropriately diluted with 50 mM phosphate buffer pH 6.8 and 100 μL was measured in a microtiter plate for optical density at 680 nm using a dual absorbance fluorescence plate reader (M1000 Infinite, Tecan, USA).

The growth rates were fit using optical density at 680 nm using the model developed by Baranyi and Roberts (1994) where N represents the cell density, *N*_*max*_ represents the maximum cell density, *μ*_*max*_ is the maximum specific growth rate, and Q is a physiological adaptation parameter used to accommodate lag time^26^:

1 $$\frac{dN}{dt}={\mu }_{max}(\frac{Q}{1+Q})(1-\frac{N}{{N}_{max}})N$$

2 $$\frac{dQ}{dt}={\mu }_{max}Q$$

### Glucose and nitrate determination

The supernatant was filtered with polyethersulfone (PES) filters and diluted at least 1:10 v/v using 5 mM H_2_SO_4_ prior to glucose and nitrate analysis by HPLC using a HiPlex H column operated under previously described conditions^27^.

### Nile red assay for lipid accumulation

Nile Red fluorescence assay was performed using bleached algae as previously described^28^. In order to improve throughput, a minor modification was made. The bleached algae were washed by transferring the cultures into a 96 well filter plate with a pore size of 0.2 μm (Millipore, USA) and centrifugation at 3500 rpm for 5 min. Residual hypochlorite was removed by mixing 1 mL of phosphate buffer (50 mM pH 6.5) to each well to wash the retained cells followed by centrifugation. This washing process was repeated 3 times. Cells were resuspended in an appropriate amount of phosphate buffer (dependent on cell concentration) and 100 μL was transferred to a clear bottom black microtiter plate. Absorbance at 680 nm was used to determine the optical dry cell weight as previously described. Following absorbance measurements, 100 μL of the Nile Red (Enzo Scientific) working solution (10 μg/mL in 50% (v/v) DMSO, 50% (v/v) 50 mM phosphate buffer pH 6.5) was added to each well. A standard lipid curve created using 5% (w/v) cream (Nelson, Canada) was included in duplicate on each plate. The plate was incubated in the dark with shaking at 40 °C and fluorescence was measured at Ex = 530 nm and Em = 570 nm after 10 min. The lipid content was determined using the following equations:

3 $$optical\,DCW[\frac{g}{L}]=\frac{2.155\times {A}_{680}}{5.363-{A}_{680}}$$

4 $$Lipid( \% wt)=\frac{F(RFU)}{b(RFU\cdot L{g}^{-1})\times optical\,DCW(g{L}^{-1})}$$

Where A_680_ is the absorbance at 600 nm using 100 μL of culture, b is the extinction coefficient for lipid concentration, optical DCW is the calculated dry cell weight of the culture prior to Nile Red addition using the optical method, and F is the relative fluorescence.

### Total lipid content by direct transesterification to fame

The fatty acid methyl ester (FAME) content by weight was determined for triplicate cultures using a slightly modified standard FAME laboratory analytical procedure (LAP) from the National Renewable Energy Laboratories (NREL)^29^. Briefly, approximately 10 mg of dried cells were mixed with 20 μL of the recovery standard pentadecanoic acid methyl ester (C15:0Me at 10 mg/mL), 300 μL of 0.6 M HCl, and 200 μL of a trichloromethane methanol mixture (2:1 v/v) and subsequently incubated for 1 h at 85 °C in a water bath with stirring on a magnetic hot plate at 1000 rpm. After cooling, 1 mL of hexane was added to each sample and mixed at ambient temperature at 1000 rpm. Samples were centrifuged and 450 μL of the clear top hexane phase was spiked with 50 μL of the internal standard undecanoic acid methyl ester (C11:0Me) to have a final concentration of 100 μg/mL. FAME was separated and analyzed using a flame ionizing detector (FID) equipped Agilent 7890 Series GC and an Agilent DB-Wax capillary column (30 m, 0.25 mm, 0.25 μm). Helium was used as the carrier gas at a constant pressure of 119 kPa, and the FID was operated at 280 °C. Samples were injected in split mode with a 1:10 split ratio and eluted using the following oven ramp: 50 °C, 1 min, 10 °C/min to 200 °C, 3 °C/min to 220 °C, and hold for 10 min. Individual FAMEs were quantified using analytical standard mixture (Supelco 37, Sigma Aldrich) and the internal standard. Unidentified FAME were quantified by applying the RF factor of the closest known peak. Total FAME content by weight was calculated according to the NREL LAP by adjusting the cumulative FAME mass using the recovery standard C15:0Me and dividing the total by the weight of cells used in the assay.

## Results and Discussion

Previous work using a standard central composite design for the optimization of glucose, nitrate, phosphate, magnesium, calcium and iron concentration was found to have poor prediction ability at the extremes resulting in significant lack of fit. Analysis of the factorial design indicated no significant effect of phosphate, calcium, and iron on maximum optical density and end point lipid content under the conditions studied (data not shown). Therefore, in order to reduce the number of runs, have all points within the design space, and improve prediction in the extremes for the remaining factors, glucose, nitrate, and magnesium, a Box-Behnken design (BBD) was chosen to gain greater insight into the interactions and effects of these factors on mixotrophic growth performance and culture productivity.

Several measurements of culture performance were determined every 12 h such as optical density, glucose consumption, as well as lipid accumulation. Lipid accumulation was measured using the Nile Red lipid assay in shaker flasks during the cultivation period as it uses small sample sizes and the end point total lipid content was confirmed using an NREL Laboratory Analytical Procedure^29^. End point lipid content determined using the NREL LAP showed reasonable correlation with the end point lipid content as determined using the Nile Red method (R^2^ = 0.7463). Optical density which is particularly prone to interference by cellular chlorophyll content was used for continuous monitoring during the cultivation period and dry cell weight measurement was conducted only at the culture end point for a more accurate determination of cell density as it requires a substantial sample size.

### Box-Behnken design response surface polynomials

**Figure 1:**
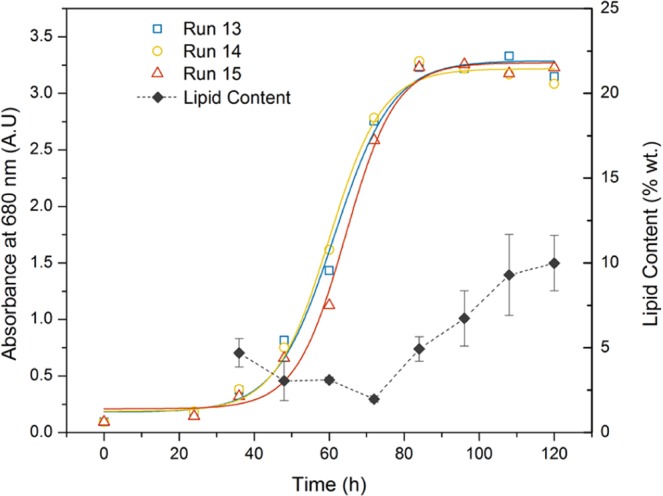
Growth and lipid content (Nile Red Assay) profiles for centre point replicates (runs 13–15) discrete data (open symbols) and their best fit according to the Baranyi model^26^ as determined by least squares non-linear regression (corresponding coloured line).

The overall goals of this optimization was to maximize the growth rate and volumetric lipid productivity in order to achieve a high overall lipid productivity (g/L·d). Single factor optimization of solely environmental factors for other species such as *Desmodesmus sp*. F2 have resulted in increases in lipid productivities as high as 2.3 fold^30^. Growth rate was determined in all 15 shaker flask runs by optical density and fit to a logistic growth model^26^ using nonlinear least squares regression in MATLAB (MathWorks, USA). Optical density data was collected every 12 h and the fitted parameters are summarized in Table 2 along with media composition, glucose consumption rate, end point DCW, end point lipid content, and volumetric lipid productivity. A comparison of the fitted regression curves and collected data and the lipid accumulation according to the Nile Red assay are illustrated in Fig. 1 for the three centre point replicates. The high variability of the lipid content in the early phase of cultivation is due to the low cell densities found in these samples.

**Table 2:** Media composition of BBD and culture performance parameters measured in this study.

Run	Glucose (g/L)	NaNO_3_(g/L)	MgSO_4_ 7H_2_O (g/L)	Baranyi Fit Parameters (A_680_)		Glucose Consumption Rate; Ω_Glc_ (g/L·h)	Nitrate Consumption Rate; Ω_Nit_ (g/L·h)	End point DCW (g/L)	End Point Lipid Content (% wt.)	Volumetric Lipid Productivity (g/L)
				μ_max_ (h^−1^)	N_max_ (A.U)					
1	2	0.23	1.38	0.227	0.773	−0.0375	−0.0147	1.15 ± 0.01	26.3 ± 3.7	0.30 ± 0.04
2	20	0.23	1.38	0.179	0.749	−0.1984	−0.0108	1.59 ± 0.02	30.2 ± 1.4	0.48 ± 0.02
3	2	2.55	1.38	0.119	1.141	−0.0292	−0.0259	1.10 ± 0.02	10.8 ± 1.2	0.12 ± 0.01
4	20	2.55	1.38	0.116	5.277	−0.1987	−0.0255	9.54 ± 0.15	14.9 ± 1.6	1.42 ± 0.13
5	2	1.39	0.25	0.085	1.767	−0.0396	−0.0178	1.28 ± 0.03	10.8 ± 0.2	0.14 ± 0.00
6	20	1.39	0.25	0.117	3.757	−0.2126	−0.0185	8.80 ± 0.09	27.8 ± 5.7	2.44 ± 0.51
7	2	1.39	2.5	0.119	1.258	−0.0263	−0.0247	1.23 ± 0.06	14.1 ± 0.8	0.17 ± 0.02
8	20	1.39	2.5	0.130	3.513	−0.2008	−0.0227	8.08 ± 0.02	27.8 ± 3.2	2.25 ± 0.25
9	11	0.23	0.25	0.094	0.919	−0.1425	−0.0028	1.79 ± 0.05	30.0 ± 0.4	0.54 ± 0.02
10	11	2.55	0.25	0.082	3.848	−0.1402	−0.0238	4.05 ± 0.09	11.0 ± 1.2	0.45 ± 0.06
11	11	0.23	2.5	0.198	0.694	−0.0345	−0.0073	1.51 ± 0.00	23.7 ± 0.7	0.43 ± 0.11
12	11	2.55	2.5	0.117	4.007	−0.1541	−0.0335	5.07 ± 0.08	11.9 ± 1.1	0.60 ± 0.06
13	11	1.39	1.38	0.149	3.462	−0.1447	−0.0210	5.27 ± 0.06	19.3 ± 2.2	1.02 ± 0.13
14	11	1.39	1.38	0.170	3.381	−0.1555	−0.0272	5.28 ± 0.00	17.3 ± 0.5	0.91 ± 0.03
15	11	1.39	1.38	0.204	3.360	−0.1488	−0.0229	5.46 ± 0.05	18.8 ± 0.6	1.03 ± 0.03

### Growth parameters

**Figure 2:**
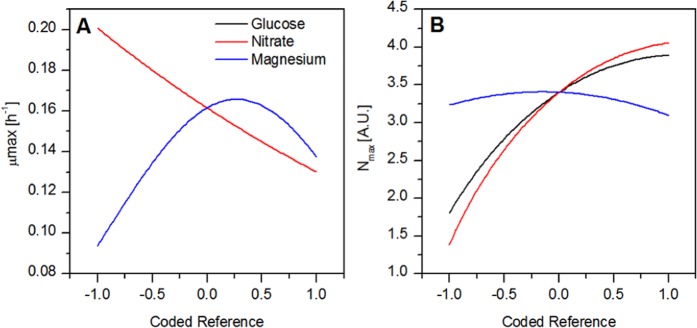
Perturbation plots of each growth parameter with respect to changes in each factor. Other factors were held at the center point (0,0) as a single factor was incremented. (**A**) Growth rate (μ_max_) (**B**) Maximum optical density (N_max_).

Analysis of variance was performed on the fitted Baranyi coefficients and is summarized for μ_max_ and N_max_ in Table S1 and S2. The other parameters fit to the Baranyi model were the initial cell concentration (N_0_) and the initial culture adaptation parameter (Q_0_) however, as these parameters are affected primarily by the initial seeding density and the cultivation conditions and media composition of the seed culture respectively, they were not investigated any further. A comparison of the actual and predicted values for the response surface polynomial fit values and *μ*_*max*_ and *N*_*max*_ are shown in Fig. S1. Perturbation plots were constructed with the resulting models in order to illustrate the relationship between each factor and the growth parameters (Fig. 2). To generate the plots, one factor level was changed while all others were held at the centre point.

**Figure 3:**
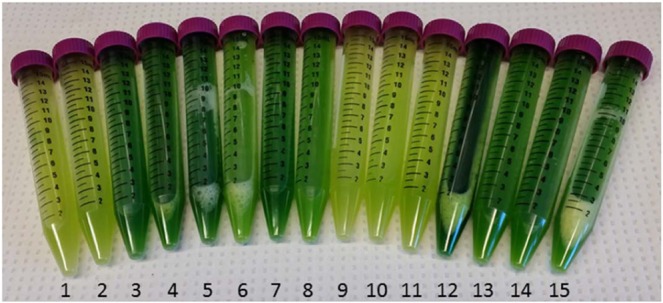
Comparison of *C. vulgaris* grown under study conditions at the culture end point. The large variation in intensity and colour is indicative of a large variation in chlorophyll content of cells grown under different conditions.

Growth rates ranged from 0.085 to 0.227 h^−1^ depending on the media composition. The lowest growth rates were experienced in cultures containing the lowest level of magnesium sulfate as is illustrated in the perturbation plot in Fig. 2A and 3.3. However, this may be due to artificially inflated absorbance in cultures which had high chlorophyll content as magnesium is an important cofactor in chlorophyll synthesis^31^. The major differences in chlorophyll content between cultures can be seen in Fig. 3. Accordingly, growth parameters fitted using optical density measurements can vary significantly from the actual dry cell weight measurements which is likely why high variance is common in investigations of microalgae cell growth. With this potential interference in mind, the highest growth rates are predicted for moderate levels of magnesium (1.5–1.7 g/L MgSO_4_·7H_2_O) and moderate levels of nitrate (1.1–1.2 g/L NaNO_3_).

The maximum optical density was found to be affected by all three factors, with glucose and nitrate concentrations exhibiting the strongest effects. Maximum optical densities occurred at the highest concentrations of glucose and nitrate (Fig. 2B and 3.3**)**. Overall, the highest maximum optical density was predicted at the highest level of nitrate (2.5 g/L NaNO_3_), the highest level of glucose (20 g/L), and moderate levels of magnesium (0.7–1.7 g/L MgSO_4_·7H_2_O). Interference of chlorophyll in the determination of maximum optical density can be seen by comparing the N_max_ to the actual DCW measurements of the culture. Run 12 which had the highest chlorophyll content as can be seen visually in Fig. 3, had a higher fitted N_max_ than run 6 which had a much higher DCW and appeared much more yellow.

### Glucose and nitrate consumption and lipid accumulation rate

Glucose consumption was monitored throughout fermentation for two purposes, firstly to determine the fermentation end point, and secondly, in order to determine which conditions if any resulted in incomplete depletion of the added glucose.

Unsurprisingly, glucose consumption rates (Table 2) were highest in the cultures containing the highest initial glucose concentration. However, ANOVA analysis using a reduced linear interactions model indicated that nitrate concentration and magnesium concentration also played a role in the consumption of glucose (Table S3). Two cultures did not consume all of the available glucose even after 168 h; culture 8 which had 1.64 g/L of glucose remaining (8.8% of starting concentration) and culture 11 which left over 55% of the original glucose unconsumed (5.53 g/L remaining). All other cultures consumed all glucose within 144 h of inoculation, however, in cultures with an initial concentration of 2 g/L, glucose was depleted within 48 h, in cultures with initial glucose concentration of 11 g/L, glucose was depleted within 72–84 h (except culture 11), and glucose depletion at the highest concentration (20 g/L) took between 96–144 h (except culture 8).

Nitrate consumption was measured (Table 2), however, it was very loosely negatively correlated to maximum cell density (N_max_, R^2^ 0.443) and positively correlated to lipid content (R^2^ 0.4989). Nitrate was not depleted in any of the cultures and remained above 0.3 g/L in all runs until growth ceased (data not shown). This is likely because nitrate concentration was higher than typically used to induce lipid accumulation, however, as a result, lipid accumulation could be achieved without limiting the maximum cell density by depleting nitrogen needed for biomass synthesis.

**Figure 4:**
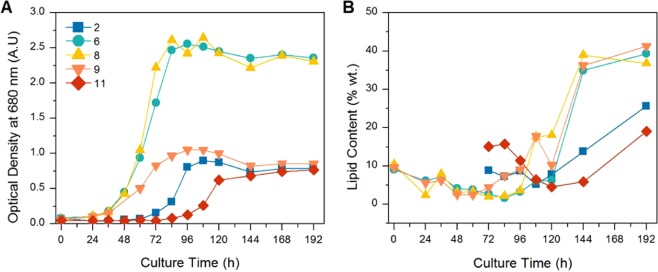
Growth (**A**) and lipid accumulation (**B**) during cultivation of selected runs as determined by the Nile Red assay.

Lipid accumulation during the cultivation was monitored using the Nile Red assay (only selected cultures are shown). Very little change in lipid content was found in most cultures, particularly those which depleted their glucose rapidly (not shown). The five cultures which did not deplete their glucose within 144 h were found to be the cultures with the highest lipid levels and lipid content increased significantly in the period after 96 h in these cultures as seen in Fig. 4. High lipid contents in the beginning of cultures 2 and 11 is likely due to the very low cell densities found in these two cultures. These results suggest that the ratio of carbon to nitrogen in these cultures was adequate for lipid accumulation to begin and that significant lipid accumulation only occurs during stationary phase. A similar phenomenon was recorded for *C. protothecoides* which only began accumulating lipids during the stationary phase^22^.

### Endpoint dry cell weight, lipid content, and lipid productivity

As previously stated, the end point DCW is a much more accurate representation of the culture performance than optical density. Similarly, the Nile Red (NR) assay is also subject to significant error^28^, therefore end point lipid content was also determined using a direct transesterification method. End point DCW was measured in triplicate for each culture and averaged **(**Table 2). The DCW was fit to a reduced cubic polynomial following ANOVA analysis (Table 3) which resulted in the following uncoded equation:

$$\begin{array}{rcl}DC{W}^{0.2} & = & 0.9634+0.0185\times [Glc]+0.0177\times [NaN{O}_{3}]+0.0362\times [MgS{O}_{4}\cdot 7{H}_{2}O]\\  &  & +\,0.0315\times [Glc]\times [NaN{O}_{3}]+0.0188\times [NaN{O}_{3}]\times [MgS{O}_{4}\cdot 7{H}_{2}O]\\  &  & -\,(9.7769\times {10}^{-4})\times {[Glc]}^{2}-0.0242\times {[NaN{O}_{3}]}^{2}-0.0231\times {[MgS{O}_{4}\cdot 7{H}_{2}O]}^{2}\\  &  & -\,(7.2155\times {10}^{-3})\times [Glc]\times {[NaN{O}_{3}]}^{2}\end{array}$$

where [*Glc*] is the concentration of glucose (g/L), [*NaNO*_3_] is the concentration of sodium nitrate g/L), and [*MgSO*_4_·7*H*_2_*O*] is the concentration of MgSO_4_ 7H_2_O (g/L).

**Table 3:** ANOVA for end point dry cell weight after a box-cox transformation of λ = 0.2 and elimination of insignificant terms using backward elimination, α = 0.1.

Source	Sum of Squares	DF	Mean Square	F-Value	*p*-value (Prob > F)
**Model**	**0.60**	**9**	**0.066**	**471.70**	**<0.0001**
*Glucose (x*_1_)	*0.23*	*1*	*0*.2*3*	*1670.16*	**<*0.0001***
*Nitrate (x*_*2*_)	*0.12*	*1*	*0.12*	*821.21*	**<*0.0001***
*Magnesium (x*_3_)	*1.764* × 10^−5^	*1*	*1.764* × 10^−5^	*0.13*	*0.7377*
*x*_*1*_*x*_2_	*0.058*	*1*	*0.058*	*414.36*	**<*0.0001***
*x*_2_*x*_3_	*2.425* × 10^−3^	*1*	*2.425* × 10^−3^	*17.23*	***0.0089***
*x*_1_^2^	*0.023*	*1*	*0.02*3	*164.59*	**<*0.0001***
*x*_2_^*2*^	*0.072*	*1*	*0.072*	*514.03*	**<*0.0001***
*x*_*3*_^2^	*3.168* × 10^−3^	*1*	*3.168* × 10^−3^	*22.51*	***0.0051***
*x*_*1*_*x*_*2*_^*2*^	*0.015*	*1*	*0.015*	*109.48*	***0.0001***
**Residual**	**7.035** × **10**^−**4**^	**5**	**1.407** × **10**^−**4**^		
*Lack of Fit*	*6.392* × 10^−4^	*3*	*2.131* × 10^−4^	*6.63*	*0.1339*
*Pure Error*	*6.429* × 10^−5^	*2*	*3.214* × 10^−5^		
**Total**	**0.60**	**14**			

**Figure 5:**
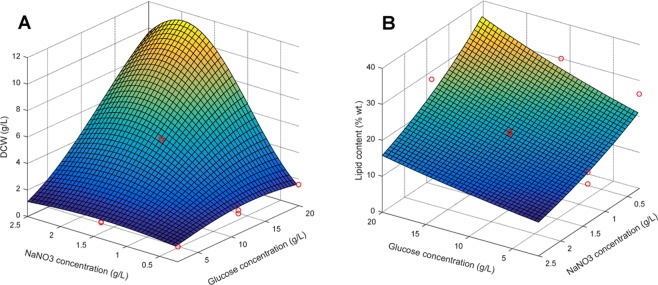
Effects of glucose and nitrate concentration on DCW and lipid content according to generated response surface polynomials. Actual data points are shown as red circles.

The DCW varied significantly from 1.1 g/L to 9.5 g/L depending on the media composition. There was excellent fit between a reduced cubic model and the data and no significant lack of fit was detected (R^2^ of 99.88% and R^2^ adjusted of 99.67%). Correlation between the predicted values and the actual values was excellent (R^2^ of prediction of 98.10%). The higher order polynomial needed in this case is a direct reflection of the complex relationship between nitrate and glucose concentrations (*x*_1_*x*_2_, *p* < 0.0001 *and x*_1_*x*_2_^2^, *p* = 0.0001). The effect of glucose and nitrate on end point DCW is illustrated in Fig. 5 which shows that the highest cell densities were achieved when nitrate was at a moderately high level (1.15–2.5 g/L) and glucose was at the maximum concentration (17–20 g/L). End point lipid content was also determined in triplicate for each culture using direct transesterification to FAME followed by quantification using GC (Table 2). End point lipid content fit reasonably well to a simple linear polynomial:

$$\mathrm{ln}(lipid\,content)=3.07856+0.029036\times [Glc]-0.35311\times [NaN{O}_{3}]$$

where [*Glc*] is the concentration of glucose (g/L), [*NaNO*_3_] is the concentration of sodium nitrate (g/L), and lipid content measured as percent dry weight.

There was reasonable fit between the data and the linear model (R^2^ of 87.66% and R^2^ adjusted of 85.60%), prediction was adequate (R^2^ of prediction of 78.45%), and there was no significant lack of fit (Table 4). End point lipid content varied significantly from 10.8–30.2% wt. depending on media composition and was found to be most highly affected by nitrate concentration with high nitrate concentrations resulting in the lowest lipid contents as expected. The highest levels of lipid accumulation can be seen in Fig. 5 and occurred at the highest concentration of glucose (20 g/L) and the lowest concentrations of nitrate (0.255 g/L).

**Table 4:** ANOVA for end point lipid content (% wt.) after a box-cox transformation of λ = 0 and elimination of insignificant terms using backward elimination, α = 0.1.

Source	Sum of Squares	DF	Mean Square	F-Value	*p*-value (Prob > F)
**Model**	**1.89**	**2**	**0.95**	**42.62**	**<0.0001**
*Glucose (x*_*1*_)	*0.55*	*1*	*0.55*	2*4.58*	***0.0003***
*Nitrate (x*_*2*_)	*1.35*	*1*	*1.35*	*60.65*	**<*0.0001***
**Residual**	**0.27**	**12**	**0.022**		
*Lack of Fit*	*0.26*	*10*	*0.026*	*7.50*	*0.1233*
*Pure Error*	*6.928* × 10^−3^	*2*	*3.464* × 10^−3^		
**Total**	**2.16**	**14**			

Lastly, the end point volumetric lipid production (g/L) was also considered as the product of DCW and lipid content. As demonstrated by the response surface polynomials generated for DCW and lipid content, maximizing these two outcomes requires some compromise as the lowest concentrations of nitrate resulted in the highest lipid contents but the lowest cell densities, and vice versa. Thus, the maximization of DCW and lipid content should mirror the optimization of volumetric lipid production. Indeed, ANOVA analysis of volumetric lipid production yields a polynomial almost identical to DCW (except the addition of the interaction term *x*_2_*x*_3_) and the highest volumetric productivities coincide with the highest DCW (data not shown).

### Optimization of media composition

**Figure 6:**
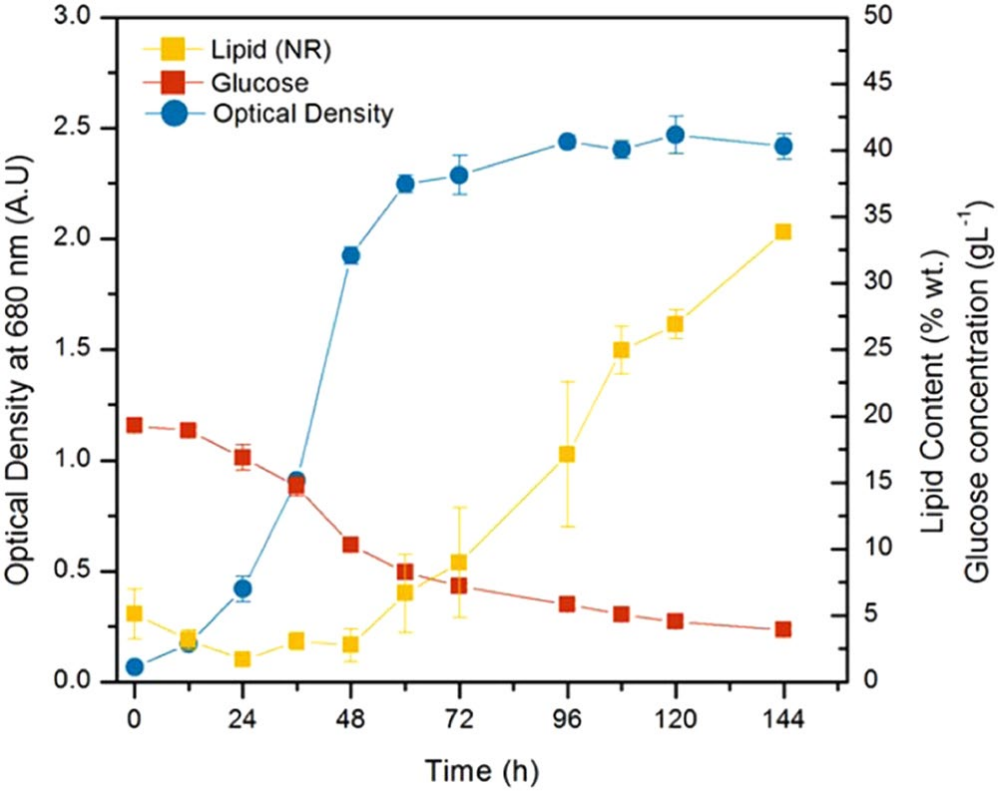
Culture performance in shaker flasks under optimal growth conditions: 18.8 g/L glucose, 1.11 g/L NaNO_3_, and 2.5 g/L MgSO_4_ 7H_2_O with light.

The media composition with respect to glucose, nitrate, and magnesium was numerically optimized using the following restrictions: the culture must achieve a cell density above 4 g/L and lipid content was maximized with a minimum content of 20% wt. With these constraints only a single general solution existed (several individual solutions within a small range), with a median of 18.8 g/L glucose, 1.11 g/L NaNO_3_, and 2.5 g/L MgSO_4_·7H_2_O. The predicted values based on the previous polynomials was compared to the experimental values obtained with this optimized media recipe in Table 5. In order to assess the quality of the prediction, these cultures were performed in triplicate in shaker flasks (Fig. 6).

**Table 5:** Comparison of prediction interval (P.I) and experimental values for the developed polynomials for [Glc] = 18.8 g/L, [NaNO_3_] = 1.11 g/L, and [MgSO_4_7H_2_O] = 2.5 g/L in shaker flask cultivations.

Parameter	P.I. (α_2_ = 0.05)	Shaker Flasks
***Baranyi Growth Parameters***		
μ_max_ [h^−1^]	0.123–0.175	0.136
N_max_ [A.U.]	2.81–3.31	2.41
***Continuous Measurements***		
Glucose Consumption Rate; Ω_Glc_ (g/L·h)	n.a*	−0.182 ± 0.02
***Endpoint Measurements***		
DCW [g/L]	5.85–6.81	6.12 ± 0.12
Lipid Content [% wt.] (NREL)	22.4–29.3	37.6 ± 2.1%
Volumetric Productivity [g/L]	1.02–2.59	2.30 ± 0.11

*Inverse transformations do not allow prediction interval calculation.

The lipid content achieved in the shaker flasks was much higher than the predicted range according to the response surface polynomial. However, it should be noted that the polynomial generated for lipid content was also found to have the poorest prediction ability, of all of the responses studied in this investigation and may be improved by additional data points as more conditions are assessed. For example, although the model fit was significant (R^2^_adj_ 0.856, Table 4) for lipid, it was not as significant as the fit for DCW (R^2^_adj_ 0.980, Table 3) and had a poorer prediction ability (R^2^_pred_ 0.784) than the DCW model (R^2^_pred_ 0.996). It was further confirmed that lipid accumulation only began with the onset of stationary phase after 48 h. Furthermore, over 10 g/L of glucose was consumed prior to the onset of stationary phase therefore an initial glucose concentration above this threshold are required in order to accumulate lipid contents above ~10% wt.

## Conclusions

In summary, the optimization of carbon and nitrogen source concentration along with magnesium concentration using a small number of runs proved to be sufficient to optimize lipid productivity for a mixotrophic culture of *C. vulgaris* in two weeks. End point measurement of lipid content using a direct transesterification process combined with dry cell weight measurement proved to be sufficient to optimizing volumetric lipid productivity. The optimal conditions in shaker flasks resulted in a high biomass density (6.1 g/L) and lipid content (37.6% wt.) after 6 days of cultivation. With minor modifications, the strategy employed here could be used for the optimization of any species of microalgae and any carbon/nitrogen source.

## Supplementary information

Supplementart Information
