# Supplementary material for: Fast media optimization for mixotrophic cultivation of Chlorella vulgaris
Source: Sci Rep. 2019 Dec 17;9:19262. doi: 10.1038/s41598-019-55870-9 (PMC6917816; doi:10.1038/s41598-019-55870-9)
Supplement: Supplementary file 1 — Supplementart Information [file 41598_2019_55870_MOESM1_ESM.docx]

Fast media optimization for mixotrophic cultivation of *Chlorella vulgaris*

Valerie C. A. Ward^a,b^ and Lars Rehmann^a^ *

^a^ Department of Chemical and Biochemical Engineering, University of Western Ontario, 1151 Richmond St., London, Ontario, Canada, N6A 3K7

^b^ Department of Chemical Engineering, University of Waterloo, 200 University Ave. W, Waterloo, Ontario, Canada, N2L 3G1.

*Email: [lrehmann@uwo.ca](mailto:lrehmann@uwo.ca)

# Supplementary Information

**Table S 1 -** ANOVA for Baranyi growth rate using optical density (μ_max_) after a box-cox transformation of λ = 0 and elimination of insignificant terms using backward elimination, α = 0.1

| **Source** | **Sum of Squares** | **DF** | **Mean Square** | **F-Value** | **p-value (Prob > F)** |
| --- | --- | --- | --- | --- | --- |
| **Model** | 1.13 | 3 | 0.38 | 15.04 | **0.0003** |
| Nitrate (x_2_) | 0.38 | 1 | 0.38 | 15.03 | **0.0026** |
| Magnesium (x_3_) | 0.29 | 1 | 0.29 | 11.57 | **0.0059** |
| x_3_^2^ | 0.46 | 1 | 0.46 | 18.52 | **0.0012** |
| **Residual** | 0.28 | 11 | 0.025 |  |  |
| Lack of Fit | 0.23 | 9 | 0.025 | 1.00 | 0.5948 |
| Pure Error | 0.050 | 2 | 0.025 |  |  |
| **Total** | 1.41 | 14 |  |  |  |

Resulting uncoded equation for factors in [g/L]

$$\ln\left( \mu_{max} \right)=-2.3234- 0.1867\times{[NaNO}_{3}]+ 0.9357\times{[MgSO}_{4}\cdot7H_{2}O] - 0.2787\times{{[MgSO}_{4}\cdot7H_{2}O]}^{2}$$

**Table S 2** – ANOVA for maximum cell density using optical density (N_max_) after a box-cox transformation of λ = 1.5 and elimination of insignificant terms using backward elimination, α = 0.1

| **Source** | **Sum of Squares** | **DF** | **Mean Square** | **F-Value** | ***p*-value (Prob > F)** |
| --- | --- | --- | --- | --- | --- |
| **Model** | 182.95 | 7 | 26.14 | 147.47 | **< 0.0001** |
| Glucose (x_1_) | 55.03 | 1 | 55.03 | 310.49 | **< 0.0001** |
| Nitrate (x_2_) | 85.31 | 1 | 85.31 | 481.38 | **< 0.0001** |
| Magnesium (x_3_) | 0.27 | 1 | 0.27 | 1.52 | 0.2579 |
| x_1_ x_2_ | 29.90 | 1 | 29.90 | 168.73 | **< 0.0001** |
| x_1_^2^ | 5.57 | 1 | 5.57 | 31.41 | **0.0008** |
| x_2_^2^ | 7.00 | 1 | 7.00 | 39.52 | **0.0004** |
| x_3_^2^ | 1.50 | 1 | 1.50 | 8.46 | 0.0227 |
| **Residual** | 1.24 | 7 | 0.18 |  |  |
| Lack of Fit | 1.20 | 5 | 0.24 | 10.79 | 0.0870 |
| Pure Error | 0.044 | 2 | 0.022 |  |  |
| **Total** | 184.19 | 14 |  |  |  |

Resulting uncoded equation for factors in [g/L]:

$${(N_{max})}^{1.5}=-1.367+0.262\times{\left[ Glc \right]+2.763 \times[NaNO}_{3}]+ 1.222\times{[MgSO}_{4}\cdot7H_{2}O] + 0.261 \times[Glc][NaNO_{3}]-0.015 \times\left[ Glc \right]^{2}-1.019 {[NaNO_{3}]}^{2}- 0.504\times{{[MgSO}_{4}\cdot7H_{2}O]}^{2}$$

**Table S 3** – ANOVA for glucose consumption (Ω_Glc_) after a box-cox transformation of (y+1)^-1^ and elimination of insignificant terms using backward elimination, α = 0.1

| **Source** | **Sum of Squares** | **DF** | **Mean Square** | **F-Value** | ***p*-value (Prob > F)** |
| --- | --- | --- | --- | --- | --- |
| **Model** | 0.11 | 4 | 0.027 | 38.70 | < 0.0001 |
| Glucose (x_1_) | 0.097 | 1 | 0.097 | 139.68 | < 0.0001 |
| Nitrate (x_2_) | 2.275×10^-3^ | 1 | 2.275×10^-3^ | 3.29 | 0.0999 |
| Magnesium (x_3_) | 2.603×10^-3^ | 1 | 2.603×10^-3^ | 3.76 | 0.0812 |
| x_2_ x_3_ | 5.592×10^-3^ | 1 | 5.592×10^-3^ | 8.08 | 0.0175 |
| **Residual** | 6.921×10^-3^ | 10 | 6.921×10^-4^ |  |  |
| Lack of Fit | 6.807×10^-3^ | 8 | 8.509×10^-4^ | 14.92 | 0.0643 |
| Pure Error | 1.141×10^-4^ | 2 | 5.703×10^-5^ |  |  |
| **Total** | 0.11 | 14 |  |  |  |

Resulting uncoded equation for factors in [g/L]:

$$\frac{1}{(\Omega_{Glc}+1)}=1.071+0.012\times{\left[ Glc \right]-0.025 \times[NaNO}_{3}]-0.056\times{[MgSO}_{4}\cdot7H_{2}O]+0.029 \times\left[ NaNO_{3} \right]{[MgSO}_{4}\cdot7H_{2}O]$$




**Figure S 1** – Parity plots for calculated and experimental Baranyi growth parameters.
